# Adhesion Improvement between PE and PA in Multilayer Rotational Molding

**DOI:** 10.3390/polym13030331

**Published:** 2021-01-21

**Authors:** Jan Sezemský, Petr Špatenka

**Affiliations:** Department of Materials Engineering, Faculty of Mechanical Engineering Czech Technical University in Prague, Karlovo Náměstí 13, 121 35 Prague 2, Czech Republic; jan.sezemsky@fs.cvut.cz

**Keywords:** multilayer rotational molding, adhesion, plasma treatment, polyethylene, polyamide

## Abstract

The aim of this study is to investigate a multilayer structure made of polyethylene and polyamide by rotational molding. Due to the different polarity of these polymers, it is difficult to ensure enough adhesion between created layers. Two methods leading to improve adhesion are introduced. Plasma modification of polyethylene powder, after which new functional groups are bound to the treated surface, may enhance specific adhesion by forming hydrogen bonds with-CONH groups of polyamide. Different strategies of adding material to the mold give rise to complicated interlayer which increases joint strength by mechanism of the mechanical adhesion. Mechanical tests show a significant improvement of joint strength, where treated samples reached two-fold values of peel strength (7.657 ± 1.024 N∙mm^−1^) against the untreated sample (3.662 ± 0.430 N∙mm^−1^). During bending test, delamination occurred only in samples that were made of the untreated polyethylene. Adding polyamide during the melting stage of polyethylene powder in rotomolding resulted in the formation of entanglements which improve the peel strength almost eight times in comparison with the sample where the polyethylene was left to completely melt and create smooth interlayer surface.

## 1. Introduction

Rotational molding is a technology used to make hollow seamless plastic products, such as various tanks, barrels and shipping containers. The principle of this technology consists of the gradual melting and sintering of polymer powder particles which cover the inner wall of the heated mold, thus obtaining the desired shape. The advantage of rotomolding is that the whole process is realized at atmospheric pressure, so the input costs are lower compared with pressure technologies. The disadvantages are the longer process times and the associated requirement for the thermal stability of used plastics. Due to this, the choice of materials is also considerably limited. The most widely used plastic in rotomolding is polyethylene (PE), which occupies up to 90% of the total production volume [1,2,3,4]. Current research in the field of the new rotational molding materials is mainly focused on the reinforcement of polyethylene, where the problem of insufficient adhesion to fibers is caused by the pressureless nature of this technology. The uniform fiber distribution across the product wall is still not fully solved as well [5,6,7,8,9,10]. Another way to expand the portfolio of materials is to combine polyethylene with other plastics, thus creating multi-layer products. However, because of the non-polar character of polyethylene it is difficult to join to other polymers and to ensure strong adhesion between them. For example, the combination of polyethylene and polyamide (PA) can be useful in producing vessels or fuel tanks. Polyamide is well known for its high permeation resistance to the hydrocarbons; therefore, it would work as an effective barrier. On the other hand, PA is an expensive polymer. However, using PE as the outer layer reduces final costs of such products and, moreover, it can protect the PA inner layer from moisture absorption [11,12]. 

In the studies [13,14] a two-layer structure was achieved in one step. The method of preparation consisted of mixing the polyethylene powder (particle dimensions were around 300 µm) and the pellets of various shapes with a size of a few millimeters. Upon heating, the smaller particles of the powder began adhering to the mold and melted first. Subsequently these particles, which created a thin outer layer, heated the pellets. However, after solidification, the inner surface of the part was not formed completely homogeneously and contained various defects. A more suitable method seemed to be the preparation of three-layer samples with an intermediate layer containing a mixture of the same powder material from which the compact outer and inner layers had been formed. In this manner, connections had been successfully made of polyethylene with polypropylene [15] as well as polyamide [16] and a thermoplastic polyurethane [17], which are completely different from polyethylene in chemical structure and their properties. The addition of an intermediate layer allowed the formation of a complex interface between the first and third layers. In studies [16,17] various factors influencing the joint strength between these layers were determined. The most important were the thickness of the interlayer and the ratio of PE in the interlayer. These factors affected the number of the entanglements, which contributed to the formation of mechanical adhesion and, as a result, to the improvement of joint strength. In [18], the investigation of the influence of particle size on the material distribution and peel strength was studied. The results showed that the powder size is important only in the formation of thin intermediate layers. The finer particles in this scale could form a greater number of entanglements that are necessary to create a mechanical interlocking. The coarser particles, which were close in size to the thickness of the interlayer, were not able to form such a complicated structure, and this resulted in lower joint strength. The coupling agents, which enable the connection of otherwise incompatible layers, are also used for adhesion improvement. This can be achieved either by mixing the base PE material with this agent, using this agent as a binding interlayer, or using chemically functionalized or grafted PE. Maleic anhydride is the most commonly used for chemical modification [12,19,20].

This article deals with the production of multilayer PE–PA samples by rotational molding technology. Because of the different chemical composition, where PE is a non-polar polymer, while PA is polar due to the presence of—CONH amide groups, there will not be enough adhesion between these two materials. The main mechanisms of adhesion between two surfaces can be accomplished by mechanical adhesion and specific (molecular) adhesion [21,22]. Mechanical adhesion, as mentioned, can be achieved in rotomolding by adding an intermediate layer formed by a mixture of the outer and inner layers. The result is an uneven interface with a larger contact area. Another proposed method to achieve mechanical adhesion during rotational molding is the following procedure. The second layer of material is added to the mold before the first layer is completely melted. Thus, a certain amount of polymer powder of the inner and outer layers is mixed directly in the mold, so improvement by mechanical adhesion is based only on a suitable strategy of adding the second layer. This is one way to improve the adhesion between the layers in this study.

The specific adhesion is achieved through the interaction of atoms and molecules. It is its strongest if chemical bonds (covalent, ionic, metallic) are formed between two surfaces. In this study, however, only weak physical intermolecular interactions between PE and PA are involved. To ensure good adhesion, it is necessary that both polymers be almost equally polar [21,23]. From the already discussed method, this includes chemical grafted or functionalized from PE. The adhesion could be also improved by plasma treatment of the polyethylene surface. During plasma modification, new functional groups (e.g., hydroxyl, carbonyl or carboxyl) are bound to the material and thus its surface energy changes [24,25,26]. Hydrogen bonds, which are the strongest intermolecular interactions, could be formed between the attached functional groups on the polyethylene surface and the—CONH groups of PA. It is on this principle that adhesion would be improved. The main advantage of plasma modification is that the changes of properties occur only on the surface, but original bulk characteristics of the material are not affected [27,28,29]. Compared to the chemical treatments, plasma modification is completely ecological.

Assumed improvement of adhesion after plasma modification is based on the several studies where a positive effect of treatment on joint strength between different materials and polymers has been reported. An example is the plasma surface treatment of fibers, which improves their wettability and thus the adhesion to the polymer matrix [30,31,32,33,34]. In the study [35], an increase up to 73% in the strength of the plasma-treated PE matrix reinforced with glass fibers was reported, compared to the matrix in the raw condition. The mechanical properties improved even when the plasma-treated PE matrix was reinforced with natural coconut fibers. The addition of new functional groups improved the hydrophilicity of PE, which could improve the compatibility between the polymer matrix and the natural fiber [9]. The combination of metals and polymers also seems promising. Even in this case, the plasma treatment positively contributes to the adhesion of otherwise incompatible materials by attaching polar groups to the surface of the untreated polymer [28,36].

## 2. Materials and Methods

### 2.1. Materials Characterization

**Figure 1:**
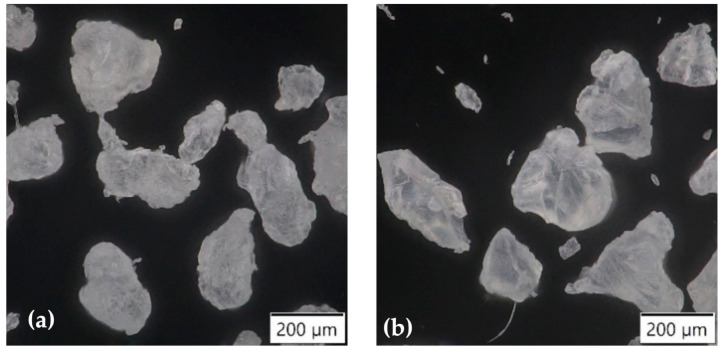
Micrographs of used powders (**a**) polyethylene; (**b**) polyamide.

Two types of rotational molding polymer powders were used in this study. The commercially available linear low-density polyethylene Dowlex™ 2629.10UE (PE) from Dow Chemical Company and the polyamide 11 Rilsan^®^ Roto 11 (PA) from Arkema company. The Figure 1 shows the micrographs of the used powders. The particles have an elongated and rounded shape with a relatively low aspect ratio. No tails, whose presence leads to high void content within the part wall, are visible. Some polymer properties provided by the producers are summarized in Table 1.

The plasma modification of polyethylene powder (TPE) was performed with an industrial scaled device LA 650 by Surface Treat, a.s., Turnov, Czech Republic. The plasma was generated by two microwave power sources with a total power up to 2 kW operating in a pulsed regime. The working atmosphere consisted of atmospheric air with a gas flow of 600 sccm. The treatment time was 9 min.

The thermal behavior of the used materials was analyzed by differential scanning calorimetry (Netzsch STA 409PG LUXX). The results showed that plasma modification did not affect the thermal properties of PE powder. The melting temperature of both untreated and treated PE was 127.5 °C, the enthalpy of fusion of untreated PE was 91.8 J∙g^−1^, plasma modified PE had the enthalpy of fusion 93.4 J∙g^−1^. Based on this result it can be assumed that untreated and treated PE will behave equally during the heating in the rotomolding process. PA 11 had a melting temperature of 187.6 °C and enthalpy of fusion 42.1 J∙g^−1^.

### 2.2. Description of the Rotomolding Machine

**Figure 2:**
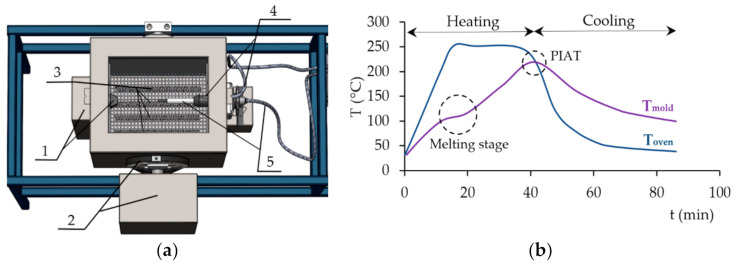
(**a**) A description of rotomolding machine 1—first stepper motor, 2—a belt drive with second stepper motor, 3—heaters, 4—temperature sensor in the oven, 5—temperature sensor in the mold; (**b**) typical curve plotted during the process.

The “rock and roll” type laboratory test machine (Figure 2a) was used for preparation of the specimen. The motion of the oven, and therefore also the mold, is ensured by two stepper motors. The first one, which is located at the front of the machine (1), provided rotation of the mold inside the oven. Its swinging is enabled by a belt drive connected to a second electric motor (2). The oven is heated by four electric resistance heaters placed at the bottom (3). Cooling is performed by a fan located underneath the oven. The temperature is measured by two sensors; the first one is in the oven (4) and the second one in the mold (5). The temperature sensor in the oven is fixed, while the temperature sensor in the mold can be pulled out of the working space. Through this gap, the next layers of material can be sequentially added to the mold during the process.

### 2.3. Specimens Preparation

The specimens were prepared in a cube-shaped aluminum form. The internal dimensions of the mold and the external dimensions of the molded specimen, respectively, are 96 × 96 × 160 mm. The temperature inside the oven was set at 250 °C. The peak internal air temperature (PIAT) was 210 °C concerning results of the differential scanning calorimetry (DSC) analysis and the experience from the article [37]. At the higher temperatures, a significant degradation of the polyamide surface occurred. Figure 2b shows the thermograph plotted during the rotational molding. When the heating cycle starts, the temperature of the air in the oven (T_oven_) increases steadily towards the set temperature. In response to the increasing oven temperature, the temperature of the air inside the mold (T_mold_) also increases. This lower curve enabled the estimate of moment at which it would be appropriate to add another layer of the material. In this study, the next layer was added during or after melting of the first PE layer. The melting stage can be identified when a visible plateau appears on the thermograph due to absorbing most of the heat input from the oven which is used for melting of the polymer particles. After this stage, the whole volume of the powder batch adheres to the mold and the majority amount of the particles are completely melted. At this point, thermal energy is directly put into to the mold again. T_mold_ has a similar rate as before melting stage and increases continuously. When the PIAT is reached, the cooling stage begins and T_oven,_ and subsequently T_mold_ start to decrease. During cooling, solidification and crystallization occur. The process cycle is completed, when T_mold_ reaches 100 °C. 

Two types of specimens were evaluated, specifically two-layer and three-layer. The prepared specimens were tested for peeling and with a three-point bend test. Samples with different thicknesses are suitable for these tests; therefore, samples intended for peeling or bending were molded from different amounts of material, as will be described below.

### 2.4. The Testing Methods

The effect of the plasma treatment and various preparation strategies were characterized by the mechanical tests and the methods of optical microscopy. The joint strength of the layers was evaluated by the peeling test, according to the modified standard ISO 11339:2010. Due to the size of the mold used, the dimensions of the test specimens had to be changed in comparison with this standard. Thus, they were cut to a length of 140 mm and a width of 20 mm. After that, the layers were separated from each other by a cross section through the center of the specimen wall to a distance of 25 mm. The specimens were clamped in the grips of the testing machine by these unbonded ends. According to the standard, a less flexible polyamide layer was attached to the movable grip. The measurement was performed on the universal testing machine MTS Exceed E42. The separation rate was set to 100 mm∙min^−1^. The peel strength (P.S.) was evaluated as the average value of force from the working diagrams by the MTS TestSuite software. Ten specimens were tested for each series.

The peel test was suitable for evaluating and quantifying adhesion between layers. However, the real components will not be stressed in this way. For this reason, a three-point bending test was performed according to ISO 178:2019. A specimen with a thickness of 5 mm was made from 300 g of raw material. The test specimens were cut to a width of 15 mm and a length of 110 mm. The flexural properties were measured by MTS Exceed E42. The distance between the supports was set to 80 mm, the test speed was 100 mm∙min^−1^. The measurement was performed by placing of the specimens on the supports with a polyamide side; the polyethylene layer was in contact with the loading edge. The flexural strength was calculated according to the equation:

*σ_fm_* = (3 *F_m_ L*)/(2 *b h*^2^), (1)

where *F_m_* is the maximum force detected during loading; *L* is the span of the supports; the width—*b* and the thickness—*h* of the specimen. The mean value was determined from five measurements. Furthermore, it was reported whether delamination of the layers occurred or not. 

The fracture surfaces after the T-peel test were optically analyzed with the digital microscope Olympus DSX1000. Some roughness parameters, namely arithmetical mean deviation of the roughness profile (Ra), mean height of the profile elements (Rc) and maximum height of the profile (Rz) were inspected on stitched images of the total surface area of 8 mm^2^ with inbuilt software. The formed interlayer was observed on 10-µm thin microtomed cross sections by polarizing microscope Nikon Eclipse ME600.

## 3. Results and Discussion

In the first experiments, the two-layer system consisting of an outer layer made from the plasma-treated polyethylene and an inner layer made of polyamide (TPE/PA) was tested. The preparation procedure was the following: a preweighed amount of material was placed in the mold. The amount of the first layer was 100 g of TPE for the peeling test samples and 150 g for bending test. The second PA layer was added after complete melting of the first layer. In this arrangement, the melting of the PE particles started when temperature inside the mold reached 96 °C and lasted for 5.1 min (in the case of 100 g) and about 7 min (in the case of 150 g). The second layer was added to the mold manually using a funnel. The amount of PA powder was identical to the first layer, i.e., 100, 150 g, respectively. After the addition of second layer, the process ran automatically. Heating continued to 210 °C, then cooling started directly inside the oven. When the temperature inside the mold reached 100 °C the machine was turned off and the mold with the finished and solidified specimen was taken out from the oven.

The preparation of the two-layer specimen improved by mechanical adhesion was based on the above basic type procedure. They differed only in the time after which the PA layer was added to the partially melted TPE layer. The labeling of these specimens was TPE/PA*X, where X indicated the time of addition of the second layer. The purpose of this preparation was to mix a certain amount of polyethylene particles with polyamide, which would create a more complicated interface after solidification. This procedure could lead to the improvement of joint strength by the mentioned mechanism of the mechanical adhesion.

Equally, the specimen composed of an outer untreated PE layer was molded. The labeling of this specimen was PE/PA*2. According to the labeling, the time of addition of second layer was 2 min after the onset of the melting of the PE particles. For longer times, the untreated PE samples were not tested, because of the insufficient adhesion between the untreated outer PE layer and the inner PA layer. The delamination had already occurred during the preparation of the testing specimens from the box-shaped specimen.

For interlayer enhancement, the three-layer system was also tested. The process parameters and the preparation of all three-layer specimens were the same as for the previous types. The difference was in the combinations of the composition of the outer layer and the intermediate layer. A total of 200 g of material was used for the peel test specimens and 300 g for the bending test specimens. The amount for each layer was equal. The intermediate layer was formed by a mixture of appropriate materials in a ratio of 1:1. The basis was the same as for the preparation of two-layer specimens. A weighed amount of the (T)PE was added to the mold, the second layer formed by a mixture of (T)PE and PA was added after melting the first layer. Subsequently, the interlayer was left in the oven for 5 min to allow the (T)PE particles in the mixture to melt. Finally, after this time, the third layer of material was added. Heating was continued to a PIAT of 210 °C, followed by cooling to 100 °C. The specimen preparation procedure is summarized in Table 2.

**Figure 3:**
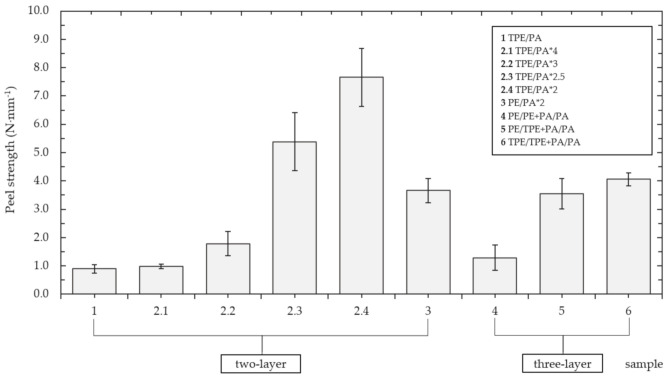
Comparison of peel strength of various samples.

The results of the peel test are summarized in the Figure 3. The basic two-layer type TPE/PA reached a peel strength of 0.897 ± 0.151 N∙mm^−1^. Gradually reducing the time of addition of the second layer, a trend of increasing joint strength is apparent. The best results had the TPE/PA*2 sample, with a peel strength of 7.657 ± 1.024 N∙mm^−1^, which is an increase of more than eight times compared to the basic type. It should be noted that the substrate (TPE) failure of this TPE/PA*2 type occurred in 8 cases out of 10. With lower adding time, the smaller amount of the polyethylene powder adhered to the mold and the rest of the loose particles mixed with added polyamide powder and together formed a thicker interlayer. However, as will be described below, the first TPE layer became thinner and therefore it was susceptible to breaking when the peel strength exceeded the strength of the base material. It could be a reason why the substrate failure occurred only in this lower-adding-time type sample. Changing the outer layer to untreated polyethylene, the peel strength of the PE/PA*2 specimens was halved to 3.662 ± 0.430 N∙mm^−1^. This indicates the plasma surface treatment has a positive effect on adhesion improvement between polymers. This effect is well known for example in adhesive joints [38,39,40,41] or modifying of polymer films and fabric [42,43,44,45]. The influence of plasma modified powder in rotational molding was examined only in this study.

The obtained results show that the value of the peel strength can also be influenced by the strategy of adding layers. The three-layer samples did not achieve the same strength values as the two-layer samples. The three-layer type PE/PE+PA/PA, consisting only of untreated PE powder, with its peel strength of 1.290 ± 0.440 N∙mm^−1^ corresponded to the basic two-layer TPE/PA. When compared to the untreated two-layer type PE/PA*2, the decrease is almost three-fold. An almost three-fold increase occurred when using the plasma-treated powder, either by adding it to the intermediate layer (PE/TPE + PA/PA) or to both layers (TPE/TPE + PA/PA). The resulting strengths of these specimens were 3.553 ± 0.540 and 4.059 ± 0.227 N∙mm^−1^, respectively. This again confirmed the assumption the use of plasma treatment would improve joint strength between PE and PA.

**Figure 4:**
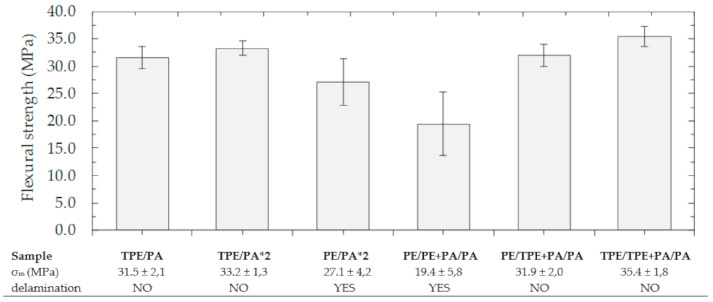
Results obtained from the bending test.

The results of the three-point bending test are shown in the Figure 4. In this case, the values of flexural strength are often more than 30 MPa. Delamination of layers was not observed in TPE/PA, TPE/PA*2, PE/TPE + PA/PA and TPE/TPE + PA/PA samples. Since the amount of used materials and their ratio were not changed, the flexural strength fluctuated around the mentioned value. The flexural strength decreased in the samples formed by untreated PE powder due to the separation of layers. For PE/PA*2 type delamination occurred in two of five samples and for PE/PE + PA/PA even in four of five samples. This behavior can be explained by the fact that various undercuts and entanglements are represented by the point contact mechanism. Thus, delamination may occur more easily if sufficiently large or numerous entanglements are not formed [46]. In contrast, after plasma modification, surface contact can be applied due to the presence of hydrogen bonds in the whole interlayer, formed between PA and bound polar groups on the PE surface. Because of that, the basic TPE/PA type also had good adhesion during the bending test, which otherwise, due to the absence of mechanical locks, did not achieve such a satisfactory peel strength [47].

**Figure 5:**
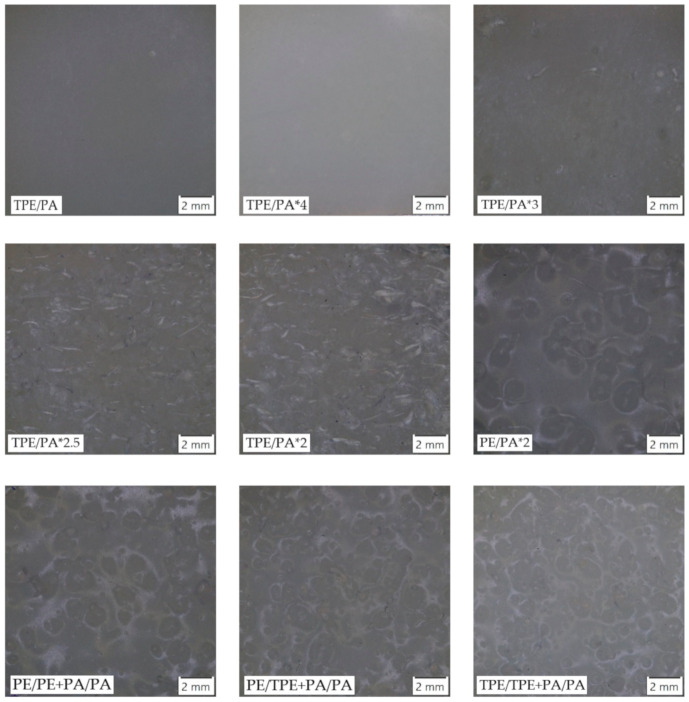
Micrographs of fracture surfaces.

The observation of fracture surfaces after the peel test is shown in the Figure 5. The results of surface roughness measurement, compared with corresponding peel strength, are reported in Table 3. The TPE/PA samples had a smooth inner surface because of the complete melting of the polyethylene layer during heating. On TPE/PA*4 no remains of entanglements were visible. So, it can be assumed that there was not enough material left to form the undercuts, because after 4 min, almost all the volume of the material melted and created a smooth inner surface. These observations were supported by results from the measurement of the roughness parameters. These two types exhibited the lowest values of Ra, Rc and Rz in a comparison with the rest of the samples. When the adding time was reduced to 3 min, the first several entanglements could be observed. With a gradual decrease in adding time, more raw PE material remained in the mold, which was mixed with the added PA during the melting process. Consequently, more undercuts were created, and the surface became rougher [48]. This trend is supported by the obtained results, where roughness of fracture surfaces increased with decreasing adding time. Two-layer samples in which mechanical adhesion appeared were formed by thin, but longer and more numerous entanglements, while three-layer samples had rounded and coarser undercuts. However, it is not possible to determine, based on obtained results, which of these two types would be better in terms of the achieved roughness parameters, as they reached similar values.

**Figure 6:**
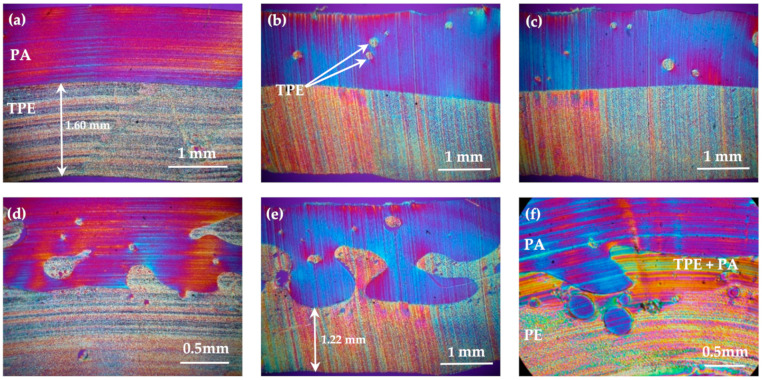
Pictures of cross-sections in polarized microscopy (**a**) plasma-treated polyethylene (TPE)/polyamide (PA); (**b**) TPE/PA*4; (**c**) TPE/PA*3; (**d**) TPE/PA*2.5, (**e**) TPE/PA*2; (**f**) PE/TPE + PA/PA.

The Figure 6 shows polarized micrographs of the cross-sections of samples’ walls. No bubbles, which are main defect when the rotomolding process parameters are incorrectly set, were detected. Unfortunately, a few cracks caused by preparation of thin film are visible.

Basic type TPE/PA (a) revealed a smooth interlayer surface. In TPE/PA*4 (b) and TPE/PA*3 (c) inner layer, the particles of PE, which mixed with PA, were found. However, that amount was not sufficient to create a more complex interlayer and undercuts, so the transition in fact remained smooth. Thus, these samples had the lowest values of the parameters Ra, Rc and Rz. This observation corresponds with the light microscopy observations. TPE/PA*2.2.d), TPE/PA*2 (e) and three-layer PE/TPE + PA/PA (f) had jagged interlayer formed by noticeable entanglements and undercuts. They functioned as mechanical interlocks and improved joint strength by the mechanism of mechanical adhesion, which corresponded with results obtained in [15,16,17]. In the TPE/PA*2 polarized micrograph, the most complicated and heterogenous interlayer with wide and high entanglements was observed. After the peel test these pulled out undercuts participated in the highest values of roughness parameters compared to the other specimen types. As already discussed, formation of a thin polyethylene layer with low adding time is visible on the cross-section, and with regard to the mentioned complex interlayer, it explains the substrate failure of TPE/PA*2 specimens during the peel test.

**Figure 7:**
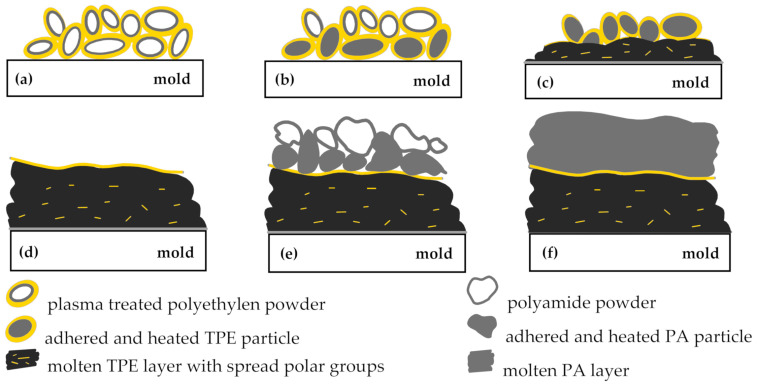
The TPE and PA layer built-up in multilayer rotational molding.

Adhesion improvement between plasma-treated polyethylene and polyamide caused by formation of hydrogen bonds is due to the specific nature of rotational molding technology, as illustrated in the Figure 7. At the beginning of the process, the TPE powder particles tumble freely in the mold (a). When the inner surface of the mold is hot enough, the powder particles adhere to the wall and stick to each other (b). After reaching the melting temperature, the particles, which are in the immediate vicinity of the mold wall, start to melt (c) and gradually form a homogenous layer of the molten plastic on the inner wall of the mold (d). The rotational molding is characterized by nearly zero shear process, with very little flow of the molten plastic. The polymeric material stays in practically the same location, where it adhered at first [2,3,17], and the surface of the molted layer is formed by surfaces of sintered TPE powder. Therefore, the upper layer of the molted plasma modified TPE particles form a thin nanolayer enriched by polar groups. If the PA powder is added to the mold during the process, the PA particles start to melt, forming the second layer (e,f). Hydrogen bonds are formed at the contact area resulting in adhesion enhancement between both layers. This mechanism was also proved by measurement of surface energy using Acrotest test inks. The surface energy of the samples sintered from non-treated PE and plasma treated TPE powders was 30 mN∙m^−1^ and more than 40 mN∙m^−1^, respectively.

It should be noted that plasma modification of TPE powders, which melted before the last TPE particles on inner surface, spreads in the molten resin and does not contribute to adhesion between the polyethylene and the polyamide layers.

Preliminary experiments were conducted to test the barrier properties of the combined PE/PA system. Our first results showed that permeation of the hydrocarbon is reduced up to 25%, when PA is used as a protective barrier layer. Additional experiments will be done to optimize the barrier properties of these sandwich specimens.

## 4. Conclusions

A new method for production of two-layer PE/PA system using rotational molding technology is presented. The system is based on the application of plasma-treated polyethylene powder for sintering the first polyethylene layer. Polar hydroxyl groups formed on the surface of the sintered TPE layer form an additional adhesion mechanism, probably based on hydrogen bonding with amide groups of the subsequent polyamide layer and improve specific adhesion between these polymers. Adding the second layer during heating and melting of the first layer leads to the creation of a more-structured interface between the outer TPE layer and inner PA layer. In this case, the joint strength was additionally improved by the combination of chemical bonding and mechanical anchoring.

## Figures and Tables

**Table 1:** Properties of used polymer powders.

Properties		Dowlex™ 2629.10UE	Rilsan^®^ Roto 11
Density	g∙cm^−3^	0.935	1.05
Melt flow index (MFI) (190 °C/2.16 kg)	g/10 min	4.0	3.8
Tensile strength	MPa	17.5	48
Flexural modulus	MPa	645	1300
Impact strength (23 °C)	J	72	65

**Table 2:** Summary of the specimen preparation. Plasma treated polyethylene (TPE), polyamide (PA), polyethylen (PE).

Type		Amount of Powder (g)			Adding Time of Next Layer (min)		Processing Parameters	
		1st Layer	2nd Layer	3rd Layer	2nd Layer	3rd Layer		
**2 layers**	TPE/PA	100; 150	100; 150	none	5.1	none	Oven temperature	250 °C
	TPE/PA*4	100	100		4			
	TPE/PA*3				3		PIAT	210 °C
	TPE/PA*2.5				2.5			
	TPE/PA*2	100; 150	100; 150		2		Rotation speed	10 rpm
	PE/PA*2				2			
**3 layers**	PE/PE + PA/PA	66 and 100	33,5 + 33,5 and 50 + 50	66 and 100	5.1	5	Rocking f.	2 cycles pm
	PE/TPE + PA/PA							
	TPE/TPE + PA/PA						Max. angle of rocking	±45°

**Table 3:** The results of roughness parameters obtained by light microscopy.

Specimen	P.S. (N∙mm^−1^)	Rz (µm)	Ra (µm)	Rc (µm)
TPE/PA	0.897	3.664	0.750	2.050
TPE/PA*4	0.984	3.161	0.559	1.438
TPE/PA*3	1.787	57.427	9.318	6.052
TPE/PA*2.5	5.387	263.615	51.745	137.241
TPE/PA*2	7.657	353.328	67.225	137.387
PE/PA*2	3.662	241.898	41.287	42.260
PE/PE + PA/PA	1.290	225.576	41.536	40.469
PE/TPE + PA/PA	3.553	140.517	25.605	30.658
TPE/TPE + PA/PA	4.059	350.452	61.186	68.037

## Data Availability

The data presented in this study are available on request from the corresponding author.
